# Untargeted Metabolomics Pilot Study Using UHPLC-qTOF MS Profile in Sows’ Urine Reveals Metabolites of Bladder Inflammation

**DOI:** 10.3390/metabo12121186

**Published:** 2022-11-28

**Authors:** Petros Pousinis, Christina Virgiliou, Thomai Mouskeftara, Sofia Chalvatzi, Fotios Kroustallas, Eleftherios Panteris, Georgios A. Papadopoulos, Paschalis Fortomaris, Michaela Cernat, Leonidas Leontides, Olga Begou

**Affiliations:** 1 Department of Chemistry, Aristotle University of Thessaloniki, 54124 Thessaloniki, Greece; 2 Biomic AUTh, Center for Interdisciplinary Research and Innovation (CIRI-AUTH), Balkan Center B1.4, 10th km Thessaloniki-Thermi Rd., 57001 Thessaloniki, Greece; 3 FoodOmicsGR, AUTh Node, Center for Interdisciplinary Research and Innovation (CIRI-AUTH), Balkan Center B1.4, 10th km Thessaloniki-Thermi Rd., 57001 Thessaloniki, Greece; 4 Laboratory of Animal Husbandry, Faculty of Veterinary Medicine, School of Health Sciences, Aristotle University of Thessaloniki, 54124 Thessaloniki, Greece; 5 Department of Epidemiology, Biostatistics and Economics of Animal Production, School of Veterinary Medicine, University of Thessaly, 43132 Karditsa, Greece

**Keywords:** metabolomics, sows, urine, urinary tract infections, biomarkers, LC-MS, mass spectrometry

## Abstract

Urinary tract infections (UTI) of sows (characterized by ascending infections of the urinary bladder (cyst), ureters, and renal pelvis), are major health issues with a significant economic impact to the swine industry. The current detection of UTI incidents lacks sensitivity; thus, UTIs remain largely under-diagnosed. The value of metabolomics in unraveling the mechanisms of sow UTI has not yet been established. This study aims to investigate the urine metabolome of sows for UTI biomarkers. Urine samples were collected from 58 culled sows from a farrow-to-finish herd in Greece. Urine metabolomic profiles in 31 healthy controls and in 27 inflammatory ones were evaluated. UHPLC-qTOF MS/MS was applied for the analysis with a combination of multivariate and univariate statistical analysis. Eighteen potential markers were found. The changes in several urine metabolites classes (nucleosides, indoles, isoflavones, and dipeptides), as well as amino-acids allowed for an adequate discrimination between the study groups. Identified metabolites were involved in purine metabolism; phenylalanine; tyrosine and tryptophan biosynthesis; and phenylalanine metabolism. Through ROC analysis it was shown that the 18 identified metabolite biomarkers exhibited good predictive accuracy. In summary, our study provided new information on the potential targets for predicting early and accurate diagnosis of UTI. Further, this information also sheds light on how it could be applied in live animals.

## 1. Introduction

The urinary tract infections (UTI) of sows, which usually involve cystitis (inflammation of the urinary bladder), ureteritis (inflammation of the ureters), and/or pyelonephritis (inflammation of the renal pelvis) can impact the health and welfare of modern sows, as well as adversely affect the economics of the swine industry worldwide. UTI has been associated with sudden death and suboptimal reproductive performances [1,2,3]. Recently, UTI has been associated with the colonization of the normally sterile urinary tract with bacteria, which are predominantly members of the fecal microbiota [4]. Bacteriuria (culture positive urine), however, may not be always detected in urine samples from sows with UTI, or, indeed, may be detected from sows without UTI [1,5]. Furthermore, other urine tests that can be performed on the live animal to diagnose UTI, such as the reagent strip tests that have been applied in some on-farm studies are ineffective, especially in the diagnosis of chronic UTI [5]. Thus, in modern swine practice, UTIs remain largely under-diagnosed and thus untreated [1,6], despite being associated with several periparturient clinical problems that may lead to a producer’s decision for the early culling [2,3,7] of certain sows. In regard to dead sows, histopathology is considered the “gold standard” for diagnosis. When compared to the “gold standard”, methods diagnosing UTI in live animals demonstrated low sensitivity but relatively high specificity in detecting UTI [5]. Therefore, alternative, rapid, robust, and reproducible methods exhibiting high sensitivity, specificity, and prediction accuracy for the purposes of detecting UTI (and more specifically cystitis) at early stages where antibiotic treatment is still responsive, are urgently needed.

Metabolomics employing liquid chromatography high-resolution mass spectrometry (LC-HRMS) methods are currently the predominant approach for disease biomarker discoveries. Currently, they are presenting with unparallel sensitivity [8,9,10,11]. They were applied in sow biofluids in order to monitor health, reproductive performance, and the effect of diet in sow gestation cycles. In the majority of studies, the samples analyzed involved plasma and serum [12,13,14,15,16,17,18], while there were reports for intestinal microbiota [19,20,21], feces [22], colostrum [23,24], milk [25,26,27], amniotic fluid [28,29], and fetuses [30]. Only a handful of studies investigated the sows’ urine [31,32]. Notably, even though the aforementioned publications studied sows, this is not to be confused with metabolomics on pigs’ urine where there are also only limited emerging reports [33,34,35,36,37,38]. To the best of the authors’ knowledge, untargeted metabolomics LC-HRMS methods utilized with sows’ urine to monitor cystitis have not been previously reported both in Greek herds and globally. 

Therefore, the aims of this study were to: (1) Study the untargeted metabolomics profile of sows in urine using an UHPLC-qTOF MS/MS method; (2) identify significant metabolites that account for the separation between healthy controls and animals with histologically verified cystitis using multivariate (MVA) and univariate statistical analysis; and (3) map the biological pathways that are mostly altered due to inflammation between the two groups. The results of this study will aid in understanding the underlying pathophysiological mechanisms behind the UTI of sows. Further, it may reveal new targets for the purposes of early diagnoses of UTI in live sows.

## 2. Materials and Methods

### 2.1. Study Population

The studied animals originated from one indoor, farrow-to-finish herd. Before initiation of the study the herd’s owner gave their written consent for participation. The study lasted from January 2019 until April 2020. The herd’s management complied with EU directive 2001/88/DC in regard to animal welfare. The culling of sows was decided by the manager/owner of the herd without any previous interference by the investigators. Culled sows were slaughtered in an abattoir that operated following the provisions of the Council Directive 93/119/EC for slaughtering animals without unnecessary suffering. Sampled sows were most commonly culled after weaning. According to the operated herd-management software, no sow was culled due to UTI or was treated, prior to culling, with antibiotics due to clinically suspected UTI. 

### 2.2. Sample Collection and Histological Examination

During sample collection at the abattoir, there was only minimum interference in the normal slaughtering process. In this process, sow carcasses were skinned immediately after stunning and exsanguination. We removed the urinary bladders of the slaughtered sows before evisceration in order to minimize possible contamination from gut content. Then, the neck of the bladder was fastened and its surface was surgically scrubbed. Next, mid-flow urine was collected after gently shaking the bladder. Urine samples were collected into sterile containers. Lastly, urine samples and bladders were cooled immediately using portable thermo-insulated containers with ice packs and transferred to the laboratory for analysis. In the laboratory, full thickness representative samples from the neck and the fundus of all urinary bladders were obtained and fixed in 10% neutral-buffered formalin for a minimum 48 h. Then, samples were embedded in paraffin and sectioned at 4 μm thickness. Sections were stained with hematoxylin and eosin (H&E); then, they were assessed by a trained veterinary pathologist. The distribution of lesions was defined as focal, multifocal, or diffuse [12]. Cystitis was diagnosed when at least one focal area of inflammatory infiltration was observed. 

### 2.3. Chemicals and Reagents

Acetonitrile (ACN) (LC-MS grade) and formic acid (FA) were obtained from CHEM-LAB NV (Zedelgem, Belgium). Deionized water was obtained from a Milli-Q ultra-pure grade water system (18 MΩ∙cm) (Millipore, Bedford, TX, USA) (LC-MS grade).

### 2.4. Sample Preparation

Urine samples (50 μL) were left to thaw at room temperature followed by 2× dilution with ultrapure water. The samples were then centrifuged for 10 min at 4 °C and 11.800 g. The supernatant was transferred to a silanized vial for the purposes of LC-TOF-MS/MS analysis. A pooled sample was prepared as a representative quality control sample by mixing equal volumes of each urine sample [39,40,41]. Then, diluted QCs (1:2, 1:4, 1:6, and 1:8 in H_2_O) were also analyzed in order to evaluate dilution integrity of the detected features.

### 2.5. UHPLC-qTOF MS/MS Analysis

Chromatography was performed on an Ultra HPLC Elute system with an ACQUITY HSS T3 (2.1 × 100 mm) (Waters Ltd., Elstree, UK) equipped with an Elute autosampler, thermostated at 10 °C. The stationary phase and mobile phase conditions, previously reported by Lewis et al. [42] were also adopted. The binary mobile phase system consisted of solvent A: water containing 0.1% FA, and solvent B: acetonitrile 0.1% FA. The separation was performed using the gradient elution program, which is presented in Table S1. At 12.15 min the system returned to initial conditions, where the column equilibrated for 2.45 min prior to the next injection. The needle was washed with a strong wash solvent 90%, ACN 10%, and H_2_O (500 μL); as well as weak wash solvent 90%, H_2_O 10%, and ACN (500 μL) before and after each injection. The injection volume and column temperature were set at 5 μL and 50 °C, respectively.

A TIMS TOF mass spectrometer (Bruker, Bremen, Germany) in positive and negative ionization mode, was used in MS data acquisition, according to the following instrument settings: capillary 4.5 kV (+ESI) 3.2 kV (−ESI), dry temperature 220 °C, dry gas 10 L/min, and nebulizer 2.0 Bar. The data were acquired using a data dependent acquisition (DDA) mode with the range 50–1300 *m/z*, as well as a fixed spectra acquisition rate for MS/MS data at 10 Hz. Collision energy was set at 15V for precursor ions below 100 *m/z*, 25 V for precursor ions with *m/z* ranged from 100 to 500, and 40 V for precursor ions with *m/z* ranged from 500 to 1000 *m/z*, with a 0.5 s cycle time. Tune parameters were adjusted, transfer parameters were optimized, Funnel RF 1 was set to 150 Vpp and 2 was set to 200 Vpp, isCID energy 0 eV, Multipole RF 50 Vpp, and deflection delta 70 V. Quadrupole parameters, ion energy, and low mass were set to 4 eV and 90 *m/z*, respectively. In collision cell parameters, collision energy was set at 7 eV, collision RF was set at 600.0 Vpp, ions’ transfer time was set at 50 µs, and pre-pulse storage was set at 5 µs. The TOF was calibrated prior to each analysis with sodium formate at a concentration of 10 mM. In addition, during each injection between 0.1 and 0.3 min of analysis took place with a flow rate of 60 µL/h. 

### 2.6. Data Analysis

UHPLC-RP-qTOF MS/MS data were obtained using Data Analysis 5.2 (Bruker). The raw LC-MS/MS data files were first converted to .abf files using Reifycs Abf Converter (ver. 4.0.0). The converted LC-MS/MS data were then processed in MS-DIAL (ver. 4.48) [43,44] for peak picking, deconvolution of high energy spectra, feature alignment, and matching spectra from imported spectral libraries (i.e., metabolite identification). The integrated identity confirmation workflow considers accurate mass, isotope abundances, as well as fragment ion and retention time information [44]. Values were reported as peak intensity (area). Further, detailed settings for the MS-DIAL are given in the Section 2.7.

A two-tailed t-test, with unequal variance and a threshold of *p* < 0.05, was performed in Microsoft Excel. Further, a principal component analysis (PCA) was performed with SIMCA 14.0 (UMETRICS AB Sweden) via UV scaling. Data were further processed by a partial least squares discriminant analysis (PLS-DA). From these, only features with VIP value > 1 and *p*-value < 0.05 were considered. Models were then evaluated concerning the goodness of fit in the X (R2X) and Y (R2Y) variables, as well as predictability (Q2YCV), which was investigated via permutation and CV ANOVA analysis. Furthermore, in order to validate the statically significant metabolites, curve–receiver operating characteristic (AUC–ROC) curves were also calculated from the respective Monte Carlo cross-validation prediction, while using the online web software MetaboAnalyst 5.0(Montreal, Canada). In addition, based on the identified biomarkers, urine metabolomic pathway analysis was performed using the metabolic pathway analysis (MetPA), a tool in MetaboAnalyst 5.0 for the purposes of biochemical pathway annotation [45]. Additionally, GraphPad Prism 8.0 for Windows (GraphPad Software, La Jolla, CA, USA,) was used for the illustration of box-plot graphs. 

### 2.7. MS-DIAL

The settings for the non-targeted peak picking and feature alignment in MS-DIAL were as follows: Mass accuracy was set to 0.01 Da for MS1 and 0.05 for MS2 level data for the purposes of data collection and alignment. Features in the mass range of 50–1300 *m/z* and a retention time range from 0 min to 15 min with charge states +1 and +2 were accepted. Further, a minimum peak height of 10,000 counts was used in order to filter out unwanted noise. Data were smoothed using a linear moving average filter with a box size of 3 and a minimum peak width of 5 datapoints. Protonated species, sodium adducts, ammonium adducts, and water losses were considered as ion species for peak picking in the positive ion mode. This is while deprotonated species, formic adducts, and water losses were considered for the negative ion mode. Moreover, data were aligned with a retention time tolerance of 0.1 min and a mass tolerance of 0.015 Da.

For the purposes of identity confirmation workflows, features detected in all urine extract samples assessed with MS-DIAL were annotated via searching the deconvoluted fragment spectra against positive and negative mode LC-MS/MS spectra from metabolomics libraries (MSMS-Public-Pos-VS15; MSMS-Public-Neg-VS15). Mass tolerances of 0.01 Da and 0.05 Da were used on MS1 and MS2 levels, respectively. Further, an identification score cutoff of 80% was applied. 

## 3. Results

### 3.1. UHPLC-qTOF MS/MS Untargeted Profile

Principal component analysis (PCA) and partial least squares discriminant analysis (PLS-DA) were employed in order to visualize the LC-MS dataset and display the similarities and differences among the samples contained in this study. The metabolites selected for further statistical evaluation were present in 50% of the samples analyzed. In addition, they had a relative standard deviation (RSD) < 30% of the peak areas in the QC samples [A5–A7]. Analytes with higher values of RSD were excluded based on the analytical criteria for the system stability. Thus, a total of 2319 out of 2563 positive mode ions, as well as 2813 out of 3120 negative mode ions in the RP-UHPLC-qTOF MS/MS analysis of urine untargeted profiles passed quality control criteria and were selected for further data analysis. Additionally, the PCA scores’ plot of QC samples using SIMCA 14.0 was built to assess the systems’ suitability (Figure S3). 

**Figure 1:**
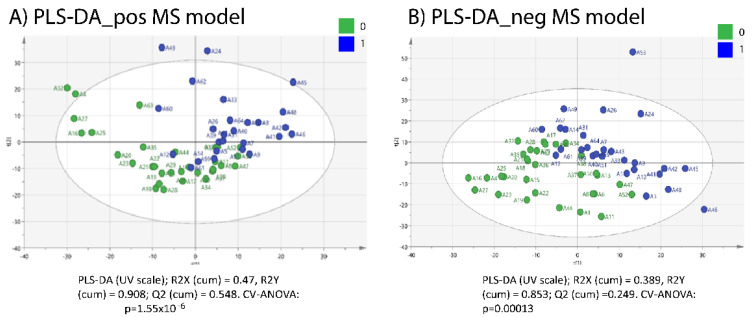
CV-validated PLS-DA models/score plots in (**A**) positive ion mode, and (**B**) in negative ion mode showing adequate separation between the two groups (healthy = 0, in green and inflammation = 1, in blue) based on their untargeted metabolomics profile in sow urine. Each dot represents a sow urine sample (n = 58 in total).

Urine samples from the 31 healthy control (CON) sows and the 27 inflammatory (INFL) sows were analyzed in both positive and negative ionization mode. Representative base peak chromatograms (BPC) from the QC samples were shown in Figure S1. Additionally, the BPC of metabolomics profiles between an INFL versus CON representative sample for the purposes of positive and negative ionization mode are given (Figure S2). As can be seen, different metabolomic profiles between a control sample when compared with an inflammatory one were observed in both ion modes; furthermore, a more prominent difference was revealed in positive ion mode. The figure depicts selected traces of two extreme samples but as a rule the majority of samples exhibited similar TICs. Therefore, no significant difference between samples was observed in unsupervised PCA analysis when features with RSD < 30% of peak areas in QCs were used. In order to further investigate the discrimination between CON sows and INFL sows, a supervised PLS-DA analysis was performed. The PLS-DA score’s plot (Figure 1A,B) shows separation between the CON sows and INLF sows in both positive and negative modes. The PLS-DA parameters were as follows: R2X (cum) = 0.47, R2Y (cum) = 0.908, and Q2 (cum) = 0.548 in positive mode; as well as R2X (cum) = 0.389, R2Y (cum) = 0.853, and Q2 (cum) = 0.249 in negative mode (Figure 1A,B). The CV-ANOVA values were *p* = 1.55 × 10^−6^ and *p* = 0.00013 for positive and negative ion mode, respectively, indicating that the models are valid (Figure 1A,B).

### 3.2. Identification of Different Metabolites

After this, an untargeted high-resolution mass measurement of molecular species and their fragmented ions, followed by a combination of MVA and univariate statistical analysis, found a total of 18 different urine metabolites (2 unknowns) from the CON and INFL sows. These have been identified and are listed in Table 1. In addition, the box and whisker plots of these 18 potential biomarkers are given (Figure S4). Generally, the results showed that the main differences between CON and INFL sows were the alteration of nucleosides (6); indoles and derivatives (2); isoflavones (2); amino acid (1); dipeptides (1); and others (4). When compared with CON sows, INFL sows increased the levels of 7 urine metabolites, as well as decreased the levels of 11 urine metabolites. All nucleosides (guanosine, adenosine, xanthosine, N2-methylguanosine, 1-methylguanosine, and cytosine) were decreased in the urine of INFL when compared to CON (see Table 1). Results showed that INFL sows had a lower level of glycyl-L-proline, 3-Methyloxyindole, and phenylalanine when compared with CON sows. On the contrary, INFL sows presented an increase in the levels of 2-Hydroxybenzaldehyde; 4-hydroxybenzoate; caffeic acid; tryptophanol; daidzein and genistein (isoflavones); and piscidic acid when compared with CON sows. Two markers of high significance are reported as unknowns due to the fact that the identities suggested (hits) from MS-DIAL could not account for their chromatographic elution (retention order) in reversed phase (RP-) chromatography (which was used in this study).

Notably, 16 out of 18 metabolites were found as potential biomarkers. These reached “level 2” identification, while 2 metabolites reached “level 5”, according to MSI guidelines by Schymanski et al. [46]. A representative depiction of MS/MS experimental spectra against reference MS/MS spectra, as outlined in MS-DIAL (v.4.48), is given for 2 metabolites: adenosine in positive ion mode, and guanosine in negative ion mode (Figure S5).

### 3.3. Prediction and Diagnostic Performance Test

**Figure 2:**
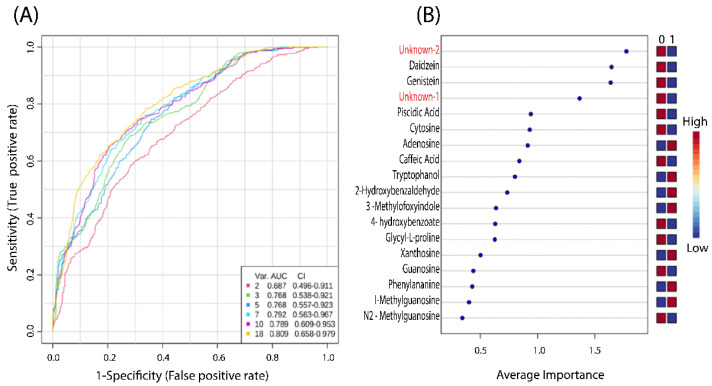
The comparison of different variables, as based on ROC curves. (**A**): The legend shows the feature numbers and the AUCs of the six models and (**B**): the average importance of 18 metabolites based on the ROC curves, in descending order of importance. The prediction of the model depends on the area under the curve (AUC) as provided by the ROC analysis—in other words, the greater the AUC, the better the prediction of the model.

ROC analysis was performed in order to validate the PLS-DA analysis and test the applicability of the 18 identified metabolite biomarkers in separating CON and INLF sows (Figure 2A,B). Figure 2A shows a group of ROC curves for models established by using different metabolites selected by the filter approach. Six models were generated. When all 18 metabolites were used, the AUC value was 0.809, while sensitivity was 74.1%, specificity 71%, and predictive accuracy was recorded at 71.8%. Based on the selected biomarkers, ROC analysis revealed that the PLS-DA model identified metabolites’ biomarkers, sorted by their importance (Figure 2B). It also accounted for the differences between CON and INFL sows.

### 3.4. Metabolic Pathway Analysis

**Figure 3:**
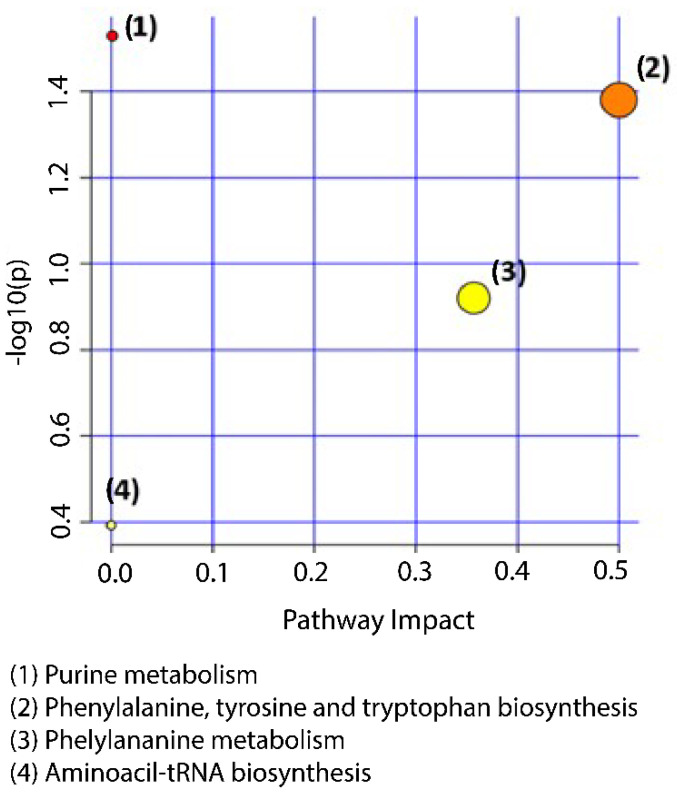
Metabolic pathway analysis of statistically significant metabolites between “healthy” and “inflammation” groups, combined in positive and negative ion mode. The pathways depicted are listed from 1 to 4 in descending order of importance, based on a combination of both the *p*-values (y-axis) and impact (x-axis). This was all performed according to the metabolic pathway analysis (MetPA) carried out in MetaboAnalyst 5.0.

Metabolites identified as potential markers (both positive and negative ion mode combined) of bladder inflammation in sow urine were related to purine metabolism (adenosine, xanthosine, and guanosine), phenylalanine, tyrosine, tryptophan biosynthesis [L-Phenylalanine], phenylalanine metabolism [L-Phenylalanine], and aminoacyl-tRNA biosynthesis [L-Phenylalanine] (Figure 3 and Table S2). The pathways were considered as the metabolic routes most significantly altered in healthy (CON) compared to inflammatory (INFL) sows. This was based on a combination criteria of the lowest *p*-values in the y-axis, as well as the pathway impact in the x-axis. Purine metabolism was the most significantly altered pathway between the CON and INFL sows.

## 4. Discussion

It is well known that UTI represent a problem for the subsequent reproductive performance [1,2,3,7] in sows. There is a very recent study on novel protein biomarkers of UTI in children [47] and emerging studies on the biomarkers of interstitial cystitis/bladder pain syndrome (IC/BPS) [48,49,50]. These developments highlight that abnormal expressions of several urine and serum specimens, including oxidative stress biomarkers (8-OHdG, 8-isoprostane, and total antioxidant capacity) [51]; growth factor; methylhistamine; glycoprotein; and chemokine and cytokines, may all be useful as biomarkers for the purposes of IC/BPS diagnosis, as reviewed here [52]. In addition, β-defensin 2 (BD-2), a specific antimicrobial peptide in the bladder, was measured in human female urine using an enzyme-linked immunosorbent assay [53]. Furthermore, BD-2 expression was found to be 18-fold higher in patients with Hunner type IC than in patients with non-Hunner type IC. However, to the best of the authors’ knowledge, there are no metabolomics studies on sow urine in order to monitor UTIs. Hence, the knowledge in regard to pathophysiology changes still remains unknown. 

In the present study, metabolomic profiles based on ultrahigh-performance liquid chromatography high resolution/time-of-flight mass spectrometry (UHPLC-HR/TOF MS), in conjunction with MVA and univariate statistical analysis, was performed in order to elucidate the underlying mechanisms of bladder inflammation on reproductive performance. 

Furthermore, 7-methylguanosine is an endogenous methylated nucleoside found in human fluids; moreover, methylated purine bases are present in higher amounts in tumor-bearing patients when compared to healthy controls [54]. Of them, 2-methylguanosine (20%) was amongst the 17 metabolites that were responsible for a higher toxicity in patients with interstitial cystitis (IC) [55], according to the group of Parsons et al. In addition, this group identified LC-MS/MS urinary cationic metabolites in patients with IC, as well as in control subjects. Our study findings confirm the role of this metabolite in sow urine. However, the fold changes in sow urine between healthy versus inflammatory groups are small. Further, due to the fact that it is not human urine, it is not possible to directly compare the results.

Adenosine is a nucleoside that is composed of adenine and D-ribose. Adenosine or adenosine derivatives play many important biological roles. This is in addition to being components of DNA and RNA [54]. Interstitial cystitis/bladder pain syndrome (IC/BPS) is a type of chronic bladder inflammation that is characterized by increased voiding frequency and pelvic pains. The sensitization of bladder afferents is widely regarded as one of the pathophysiological changes in the development of IC/BPS. Further, there is evidence that adenosine A2a receptors are involved in regulating the sensitization of sensory afferents. Yang et al. showed that suppression of adenosine A2a receptors in bladder afferents alleviates the bladder overactivity and hyperalgesia that is caused by cyclophosphamide (CYP)-induced cystitis in rats via inhibiting TRPV1 [56]. Therefore, adenosine, which was one of the more significantly altered metabolites in our study, could be a key metabolite that binds to adenosine A2a receptor in bladder afferents, thereby affecting bladder inflammation.

Caffeic acid, also known as trans-caffeate or sodium caffeate, belongs to the class of organic compounds known as hydroxycinnamic acids. Hydroxycinnamic acids are compounds containing a cinnamic acid where the benzene ring is hydroxylated [54]. Uysal et al. investigated the protective effect of caffeic acid phenethyl ester (CAPE) on cyclophosphamide-induced hemorrhagic cystitis in rats. Pretreatment with CAPE (*p* < 0.01) resulted in a significant decrease in nitric oxide levels when compared with the cyclophosphamide group [57]. In addition, though as a traditional Chinese medicine, Marsdenia tenacissima is used in treating cystitis. Within this context, Li et al. developed an LC-MS/MS method to determine the caffeic acid amongst other components from an M. tenacissima extract in rat plasma [21], thereby highlighting the role of caffeic acid in cystitis.

Cytosine is one of the four main bases found in DNA and RNA [54]. Moreover, BK polyomavirus can lead to hemorrhagic cystitis (BKHC) in allogeneic stem cell transplantation and, therefore, to an increased morbidity. Cidofovir (CDV) is a nucleotide analog of cytosine that is active against various DNA viruses. In addition, it is used for the purposes of therapy of BKPyV-HC, as was shown by Schneidewind et al. [58]. In our study, the levels of cytosine were found to be decreased in the inflammatory group. This is in consistency with the findings of Schneidewind et al., whereby they found that a reduction in cytosine analogs could induce inflammation. Moreover, Foster et al. reported that intravesicular cidofovir appears to be safe and effective for the treatment of BKHC in pediatric patients after hematopoietic stem cell transplant (HSCT) [59], thereby supporting the beneficial role of cytosine in cystitis (i.e., bladder inflammation).

Guanosine, also known as 2-amino-inosine, belongs to the class of organic compounds known as purine nucleosides [54]. Furthermore, the group of David et al. found that a Toll-like receptor 9 (TLR9) antagonist, named cytidine-phosphate-guanosine oligodeoxynucleotide 2088 (CpG ODN 2088), decreases urinary retention and ameliorates bladder morphopathology without affecting kidney function [60]. Additionally, it was reported that oxidative stress and inflammation may be key factors in the development of bladder overactivity, as shown in an atherosclerosis-induced (AI) chronic bladder ischemia rat model [61]. According to the authors, in bladders from each group, oxidative stress markers (8-hydroxy-2′-deoxyguanosine: 8-OHdG) were quantified. The levels of oxidative stress markers were significantly higher in the AI than in the other groups. This demonstrates that guanosine-related moieties play a significant role in bladder inflammation, which supports our data that reveals guanosine as a potential biomarker of bladder inflammation.

L-phenylalanine is an alpha-amino acid. L-phenylalanine is one of 20 proteinogenic amino acids, i.e., the amino acids used in the biosynthesis of proteins. Phenylalanine is found in all organisms ranging from bacteria to plants to animals. It is classified as an aromatic, non-polar amino acid. In humans, phenylalanine is an essential amino acid and the precursor of the amino acid tyrosine [54]. The group of Kim et al. demonstrated that the metabolomics profile revealed distinct expression patterns between the controls and the patients with intersitial cystitis (IC) [62]. A random forest analysis of urine samples was conducted and it was found that L-phenylalanine, purine, 5-oxoproline, and 5-hydroxyindoleacetic acid are IC specific. Although our findings revealed that L-phenylalanine is reduced in the inflammatory group in sow urine, in our study we show that there is prior evidence for the role of L-phenylalanine in IC, as well as in, subsequently, bladder inflammation.

Piscidic acid belongs to the class of organic compounds known as phenylpropanoic acids. Piscidic acid is an extremely weak basic compound. Piscidic acid has been detected in fruits and green vegetables. This could entail piscidic acid as a potential biomarker for the consumption of these foods [54]. Phenolic compounds are important bioactive compounds identified in prickly pear peel that have important antioxidant and antimicrobial properties. Alexandre et al. showed that piscidic acid was a phenolic compound present in higher amounts in prickly pear peel [63]. In our study, piscidic acid was found to be increased in the inflammatory group. Despite this—in contrast with the aforementioned study—the direct comparison of results is not appropriate due to the fact that the biological matrix is different (i.e., pear peel versus urine). Therefore, it may be that higher levels of piscidic acid in the inflammatory group could be attributed to sow defense responses to inflammation.

Tryptophol (indole-3-ethanol) is a metabolite produced by plants, bacteria, fungi, and sponges. It is a catabolite of tryptophan, converted by the gut microbiota [54]. After absorption through the intestinal epithelium, tryptophan catabolites enter the bloodstream and are later excreted in the urine. This evidence provides a plausible support for finding this metabolite in sow urine. A recent review reports on the natural occurrence, bioactivity, and various synthetic approaches to the preparation of tryptophol and its derivatives [64].

Furthermore, amongst the pathways that were altered between the healthy group versus the inflammatory group, purine metabolism and phenylalaline metabolism were ranked as the most significant. Our finding is supported by the literature due to the fact that Kim et al. reported that women with interstitial cystitis (IC) have distinct metabolomes, thereby highlighting key metabolic pathways, such as purine and phenylalaline—which may provide insight into the pathophysiology of IC [62]. 

We also need to stress the significant metabolites identified in this study—that reflect UTIs and bladder inflammation—belong to diverse groups of compounds as this was an unbiased, untargeted LC-HRMS-based metabolomics approach that was used. Therefore, caution must be used in order to derive a model based only on them. Ideally, these potential biomarkers should be the basis for more targeted LC-MS/MS (MRMs) methods. Further, they should be used in conjunction with urinalysis results and/or reagent strip tests in order to improve the accuracy of UTI diagnosis on live animals.

Lastly, we acknowledge that our pilot study presents some limitations. The main limitation is that we used only a rather small number of animals from one farm only; therefore, an external independent validation is required in further studies. More importantly we need to state that there were differences in the diet of the sows (i.e., between the different farms or geographical regions). This could represent a confounding factor due to the fact that it is reported in the literature that diet may modify metabolomic profiles in animal and human studies [65,66,67,68]. However, our findings on significant metabolites, which are supported by the literature, indicate them to be involved in cystitis—which is, itself, the underlying cause of UTIs.

## 5. Conclusions

In summary, this study demonstrates that the metabolomic profile of sows with cystitis differentiate when they are compared to healthy controls. In addition, a panel of 18 metabolites were found as potential biomarkers. The underlying changes in metabolic pathways are associated with a disorder in purine metabolism and phenylalanine metabolism, as well as phenylalanine, tyrosine, and tryptophan biosynthesis. Further, these highlight the role of adenosine, guanosine, and L-Phenylalanine in disease pathophysiology. The metabolomic workflow was designed using cutting edge analytical techniques, such as UHPLC-qTOF MS/MS, which is a combination of multivariate and univariate statistical analysis. This was achieved in addition with prediction tools so that—based on the results of our study—we would be able to further develop validated methods for the purposes of routine screening of UTIs and bladder inflammation in order to improve the productivity efficiency in the pig industry.

## Figures and Tables

**Table 1:** A list of the 18 significant metabolites of inflammation in sow urine (farm A and n = 58) as identified by the RP-UHPLC-qTOF MS/MS and PLS-DA models. Metabolites were significant when VIP > 1 and *p* < 0.05. Biomarkers were sorted by RT (ascending).

N	MS MODE	Average RT (min)	Average Mz	Metabolite Name ^a^	HMDB ID	Fold Change (FC) ^b^	*p* Value ^c^	RSD% (QCs) ^d^
1	POS	0.7	173.0912	Glycyl-L-proline	HMDB0000721	0.4	0.022	21
2	NEG	1.2	282.0838	Guanosine	HMDB0000133	0.7	0.011	7.2
3	POS	1.3	268.1036	Adenosine	HMDB0000050	0.6	0.037	9.1
4	NEG	1.4	283.0678	Xanthosine	HMDB0000299	0.6	0.017	8.1
5	NEG	1.8	296.0996	N2-Methylguanosine	HMDB0005862	0.7	0.010	2.6
6	POS	1.8	166.0717	L-Phenylalanine	HMDB0000159	0.7	0.014	11
7	POS	1.8	298.1141	1-Methylguanosine	HMDB0001563	0.7	0.014	11
8	NEG	1.9	284.088	Unknown-1		0.6	0.003	7.2
9	POS	1.9	112.05	Cytosine	HMDB0000630	0.6	0.007	11
10	NEG	2.0	255.05	Piscidic Acid	HMDB0030809	2.0	0.034	4.6
11	POS	3.0	181.049	Caffeic acid	HMDB0001964	1.8	0.039	7.6
12	POS	3.0	148.075	3-Methyloxyindole	HMDB0004186	0.6	0.015	9.3
13	POS	3.0	144.0801	Tryptophanol	HMDB0003447	2.0	0.014	9.9
14	NEG	4.6	121.0293	2-Hydroxybenzaldehyde	HMDB0034170	1.8	0.031	4.5
15	NEG	5.0	137.0241	4-hydroxybenzoate	HMDB0000500	2.2	0.035	5.4
16	POS	5.9	255.0652	Daidzein	HMDB0003312	4.3	0.045	9.2
17	POS	6.9	271.0598	Genistein	HMDB0003217	4.6	0.048	11
18	NEG	8.1	193.0344	Unknown-2		0.2	0.010	6.2

^a^ Metabolites identified by MS-DIAL v.4.48; ^b^ fold change (FC) calculated as the ratio of group mean values (inflammation group/healthy group); ^c^ *p*-value calculated by Student’s *t*-test in Excel; and ^d^ RSD % of peak areas in QCs.

## Data Availability

Available to researchers upon request.

## Supplementary Materials

The following supporting information can be downloaded at: https://www.mdpi.com/article/10.3390/metabo12121186/s1. Figure S1: Representative base peak chromatograms (BPC) of urine QC samples in (a) positive and (b) negative ionization mode. Figure S2: Representative base peak chromatograms BPC of urine samples between a control vs. inflammatory sample in (a) positive and (b) negative ionization mode. Figure S3: PCA score plot showing the examined groups together with QC samples based on the untargeted analysis data in (a) +ESI and (b) −ESI. QC samples are shown clustering together, thereby demonstrating system stability. POS MS: R2X(cum) = 0.718, Q2(cum) = 0.271; NEG MS: R2X(cum) = 0.677, and Q2(cum) = 0.281. UV scale. Then, 0 = control (in green); 1 = inflammatory (in blue); and QCs (in red). Figure S4: Box-whisker plots of 18 biomarkers between healthy/control (CON) versus inflammatory (INFL) group. Healthy = 0 (green) and inflammatory = 1 (red). Figure S5: MS/MS spectra of two representative biomarkers as shown in MS-DIAL software. (A) adenosine in positive ion mode and (B) guanosine in negative ionization mode. Table S1: Gradient elution program used for the chromatographic separation. Table S2: Result from pathway analysis.
