# Supplementary material for: Untargeted Metabolomics Pilot Study Using UHPLC-qTOF MS Profile in Sows’ Urine Reveals Metabolites of Bladder Inflammation
Source: Metabolites. 2022 Nov 28;12(12):1186. doi: 10.3390/metabo12121186 (PMC9784506; doi:10.3390/metabo12121186)
Supplement: Supplementary file 1 [file metabolites-12-01186-s001.zip › metabolites-1960129-supplementary.pdf]

Supplementary Info

Table S1. Gradient elution program used for the chromatographic separation

LINE	TIME (min)	Flow (mL/min)	%A	%B
1	0.00	0.60	99.0	1.0
2	0.10	0.60	99.0	1.0
3	10.00	0.60	45.0	55.0
4	10.15	0.61	35.0	65.0
5	10.30	0.63	25.0	75.0
6	10.45	0.67	15.0	85.0
7	10.60	0.75	5.0	95.0
8	10.70	0.80	0.0	100
9	11.00	1.00	0.0	100
10	11.55	1.00	0.0	100
11	11.65	1.00	99.0	1.0
12	11.70	0.90	99.0	1.0
13	11.80	0.80	99.0	1.0
14	11.90	0.70	99.0	1.0
15	12.00	0.65	99.0	1.0
16	12.10	0.61	99.0	1.0
17	12.15	0.60	99.0	1.0
18	15.00	0.60	99.0	1.0

Table S2. Result from Pathway Analysis.

	Total	Expected	Hits	Raw p	"-log(P)"	Holm adjust	FDR	Impact
**Purine metabolism**	66	0.69841	3	0.0296	1.5287	1	1	0.00117
**Phenylalanine, tyrosine and tryptophan biosynthesis**	4	0.042328	1	0.041702	1.3798	1	1	0.5
**Phenylalanine metabolism**	12	0.12698	1	0.12026	0.91988	1	1	0.35714
**Aminoacyl-tRNA biosynthesis**	48	0.50794	1	0.40476	0.3928	1	1	0

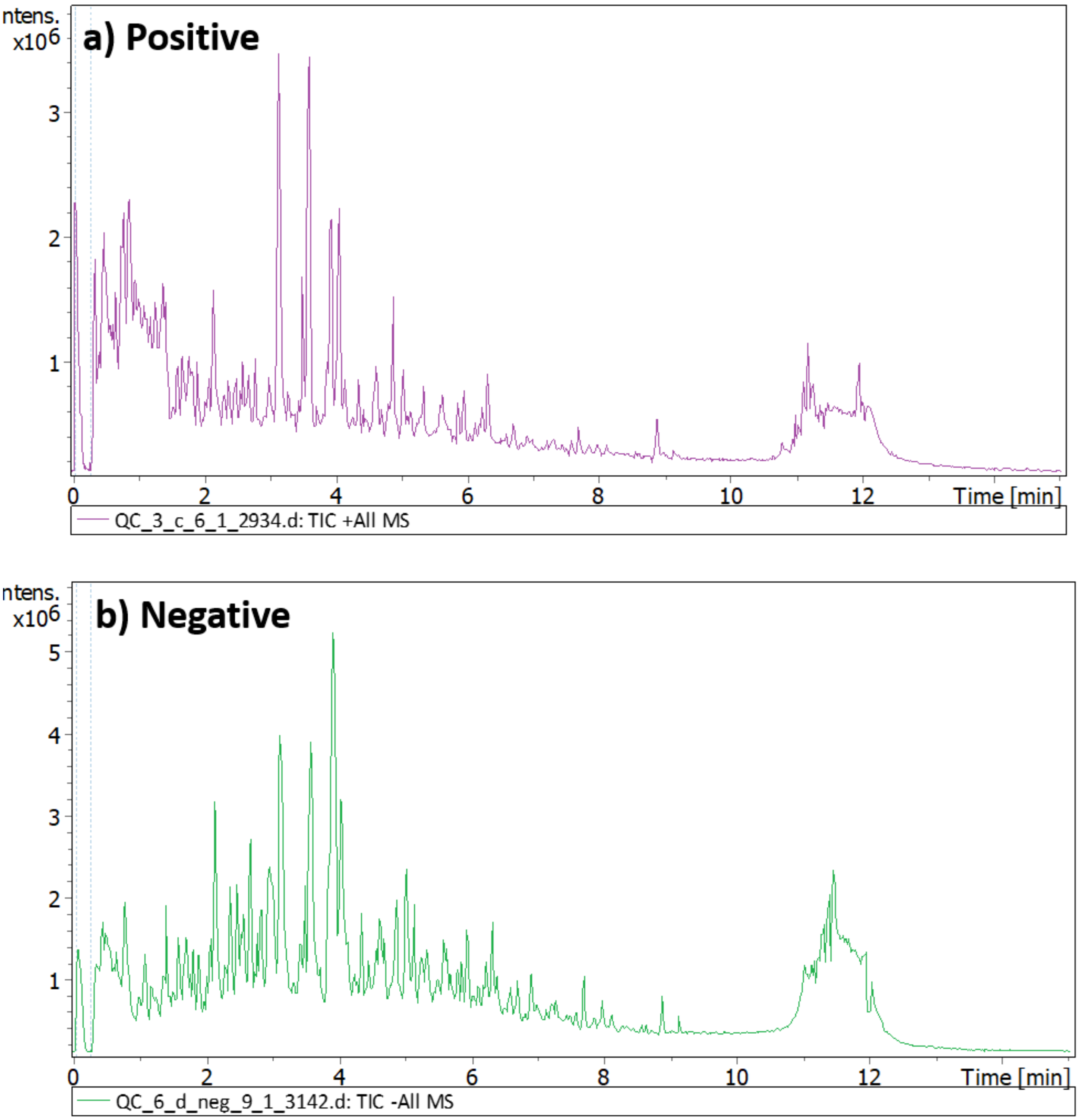

Figure S1. Representative base peak chromatograms (BPC) of urine QC samples in (a) positive, and (b) negative ionization mode.

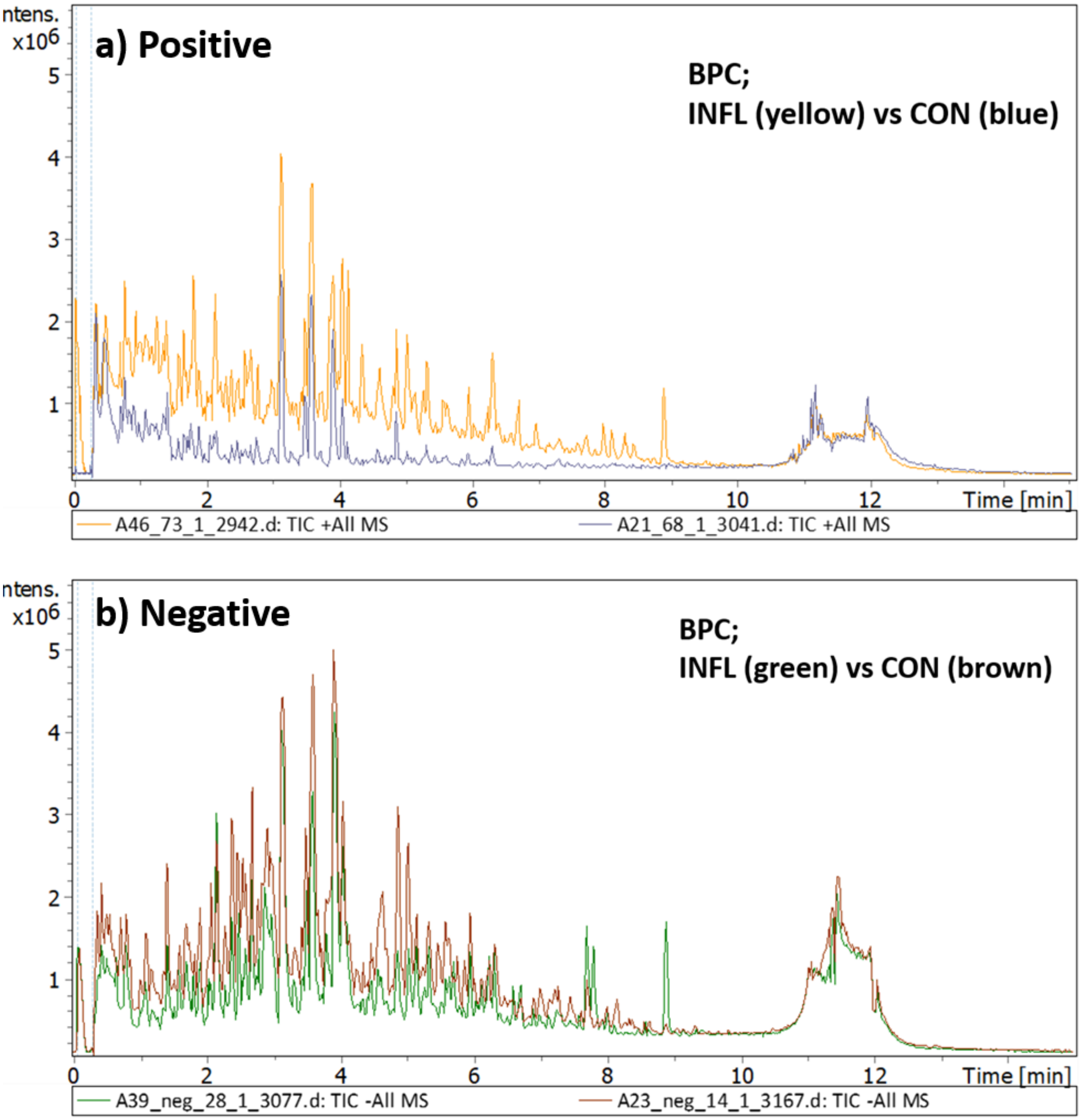

Figure S2. Representative base peak chromatograms BPC of urine samples between a control vs inflammatory sample in (a) positive, and (b) negative ionization mode.

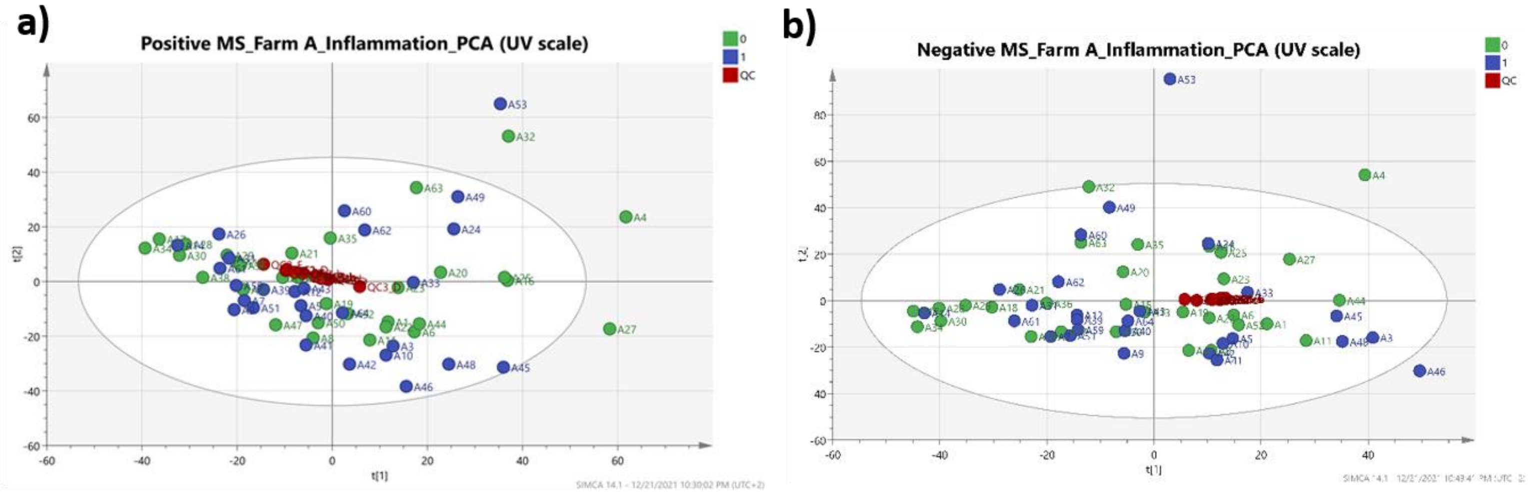

Figure S3: PCA score plot showing the examined groups together with QC samples based on the untargeted analysis data in (a) +ESI, and (b) -ESI. QC samples are clustering together, showing system stability. POS MS: R2X(cum)= 0.718, Q2(cum)= 0.271; NEG MS: R2X(cum)= 0.677, Q2(cum)= 0.281. UV scale. 0= control (in green); 1= inflammatory (in blue); QCs (in red).

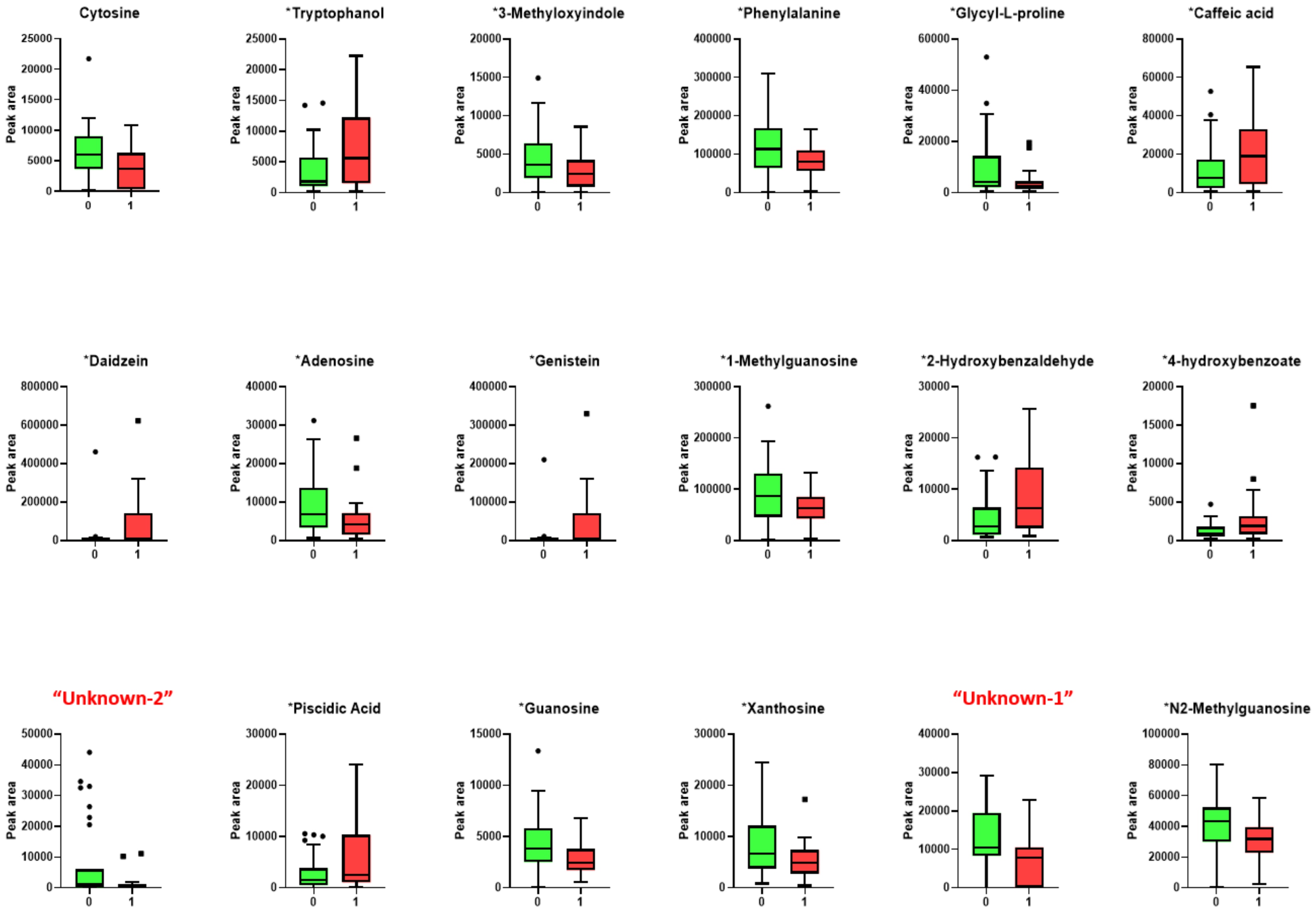

Figure S4. Box-whisker plots of 18 biomarkers between healthy/control (CON) versus inflammatory (INFL) group. Healthy=0 (green); Inflammatory=1 (red).

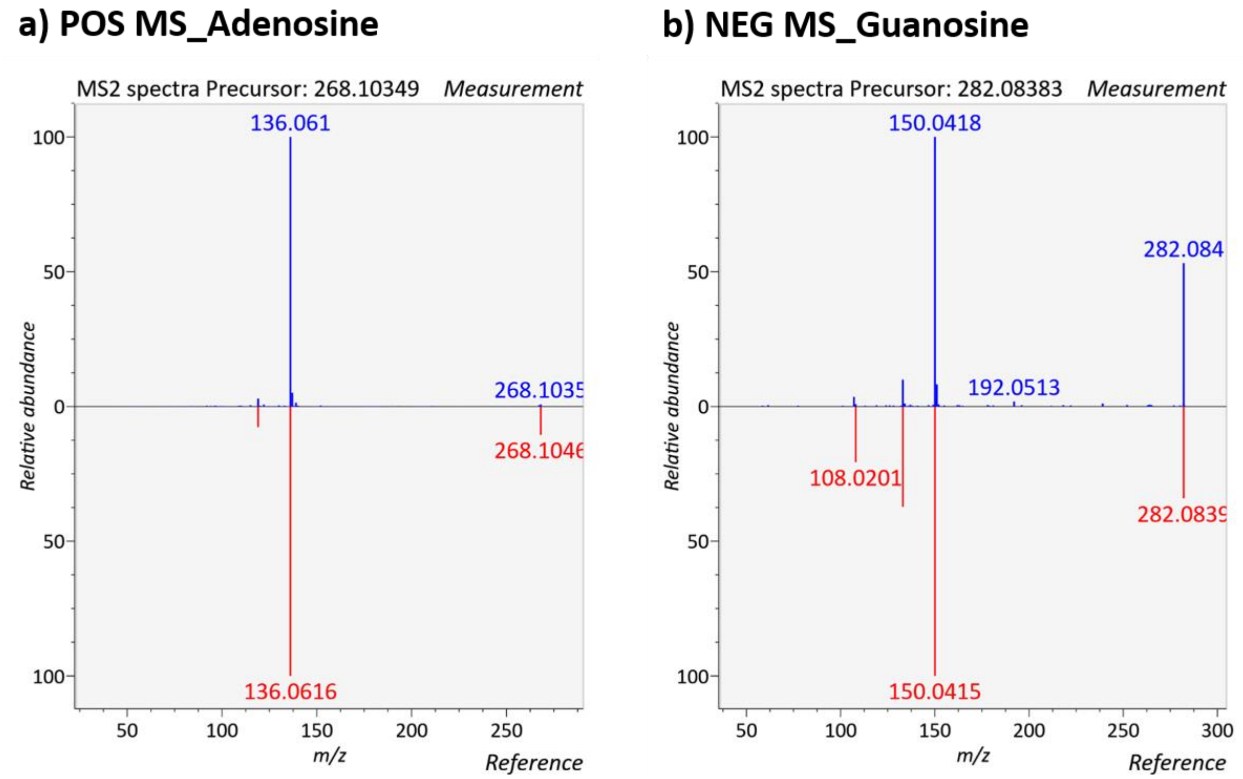

Figure S5. MS/MS spectra of 2 representative biomarkers as shown in MS-DIAL software. (A) adenosine in positive ion mode, and (B) guanosine in negative ionization mode.
